# Progressive multifocal leukoencephalopathy associated with chemotherapy induced lymphocytopenia in solid tumors – case report of an underestimated complication

**DOI:** 10.3389/fonc.2022.905103

**Published:** 2022-08-02

**Authors:** Patrick Mayr, Mathias Lutz, Maximilian Schmutz, Jens Hoeppner, Friederike Liesche-Starnecker, Jürgen Schlegel, Jochen Gaedcke, Rainer Claus

**Affiliations:** ^1^ Department of Hematology and Oncology, Medical Faculty, University of Augsburg, Augsburg, Germany; ^2^ Department of Surgery, Medical Faculty Schleswig-Holstein, University of Schleswig-Holstein, Lübeck, Germany; ^3^ Department of Neuropathology, Institute of Pathology, School of Medicine, Technical University of Munich, Munich, Germany; ^4^ Department of Pathology and Molecular Diagnostic, Medical Faculty, University of Augsburg, Augsburg, Germany; ^5^ Department of General and Visceral and Pediatric Surgery, Medical Faculty Göttingen, University of Göttingen, Göttingen, Germany; ^6^ Comprehensive Cancer Center Augsburg (CCCA), Medical Faculty, University of Augsburg, Augsburg, Germany

**Keywords:** esophageal cancer, solid tumor, JC virus, lymphopenia, (literature) review, chemotherapy, complication, progressive multifocal leukoencephalopathy

## Abstract

**Background:**

JC virus reactivation causing progressive multifocal leukoencephalopathy (PML) occurs preferentially in human immunodeficiency virus (HIV) positive individuals or patients suffering from hematologic neoplasms due to impaired viral control. Reactivation in patients suffering from solid malignancies is rarely described in published literature.

**Case Presentation:**

Here we describe a case of PML in a male patient suffering from esophageal cancer who underwent neoadjuvant radiochemotherapy and surgical resection in curative intent resulting in complete tumor remission. The radiochemotherapy regimen contained carboplatin and paclitaxel (CROSS protocol). Since therapy onset, the patient presented with persistent and progredient leukopenia and lymphopenia in absence of otherwise known risk factors for PML. Symptom onset, which comprised aphasia, word finding disorder, and paresis, was apparent 7 months after therapy initiation. There was no relief in symptoms despite standard of care PML directed supportive therapy. The patient died two months after therapy onset.

**Conclusion:**

PML is a very rare event in solid tumors without obvious states of immununosuppression and thus harbors the risk of unawareness. The reported patient suffered from lymphopenia, associated with systemic therapy, but was an otherwise immunocompetent individual. In case of neurologic impairment in patients suffering from leukopenia, PML must be considered – even in the absence of hematologic neoplasia or HIV infection.

## Background

JC virus (JCV) is an infectious agent, detectable in up to 90% of people worldwide (1). Infection is believed to occur by gastrointestinal virus uptake. In immunocompetent individuals, no evident signs of infection or concomitant disease occur, whereas in immunocompromised individuals, development of progressive multifocal leukoencephalopathy (PML) by JCV reactivation is a known complication. PML has a devastating prognosis and is routinely fatal (2, 3). Typically, individuals suffering from an infection with the human immunodeficiency virus (HIV) or from hematological neoplasms are prone to develop JCV reactivation because of prolonged lymphopenia and/or potentially insufficient lymphocyte function with reduced immunological capabilities of infection control (4). Regarding this phenomenon, many case reports and several studies have been published. Consistently, the respective authors interprete the cause of JCV reactivation in the context of lymphopenia. In clear contrast, JCV reactivation and consecutive development of PML is rarely described in solid malignancies in otherwise healthy individuals (5, 6), as long as they do not suffer from concomitant HIV infection or other immunocompromising conditions like immunosuppressive therapies such as the anti-alpha (4)-integrin antibody natalizumab (7). Only sporadic reports addressing this issue are found in current literatue, yet. Thus, awareness of PML needs to be raised in solid malignancies receiving systemic chemotherapy. Furthermore, there is a lack of studies towards clinical decision making and therapeutic options for this group of patients.

Here we report a case of JCV reactivation and consecutive fatal PML in a 69-year-old man suffering from esophageal cancer. The patient developed neurologic impairment six months after completion of neoadjuvant radiochemotherapy and surgical treatment. Initial method-dependent (mis-)interpretation of cranial computed tomography (CT) led to delay of sufficient therapy. To the best of our knowledge, no case of PML in esophageal cancer has been reported in the literature, so far.

## Case presentation

A 69-year-old male patient was diagnosed with a cT3, cN+, cM0, G2 adenocarcinoma of the distant esophagus (AEG tumor, 35 to 39 cm distal of tooth row). The patient participated in the ESOPEC trial (NCT02509286) (8), a prospective, randomized, open-labeled two-armed study comparing perioperative chemotherapy according to the FLOT protocol (fluorouracil/leucovorin, oxaliplatin and docetaxel) to neoadjuvant radiochemotherapy according to the CROSS protocol in the treatment of locally advanced esophageal cancer. The patient was assigned to the radiochemotherapy trial arm, which comprised administration of five cycles of carboplatin and paclitaxel on a weekly basis. The radiation dosage was 39.6 Gy of calculated 41.4 Gy. After an interval of six weeks, he underwent distal esophageal resection with gastric elevation according to the study protocol. By protocol, follow-up was performed by clinical assessment, by magnetic resonance (MR) imaging, and by esophagogastroduodenoscopy in intervals of three months. Until admission, the patient was free of recurrence and/or residual disease. Apart from known type II diabetes mellitus (HbA1c 52 mmol/l, upper limit of normal (ULN) 39 mmol/l) and asymptomatic sigmoid diverticulosis, the patient suffered from no other chronic, nor otherwise immunosuppressive disease or previous malignancy. The patient stopped smoking prior to AEG diagnosis.

**Figure 1:**
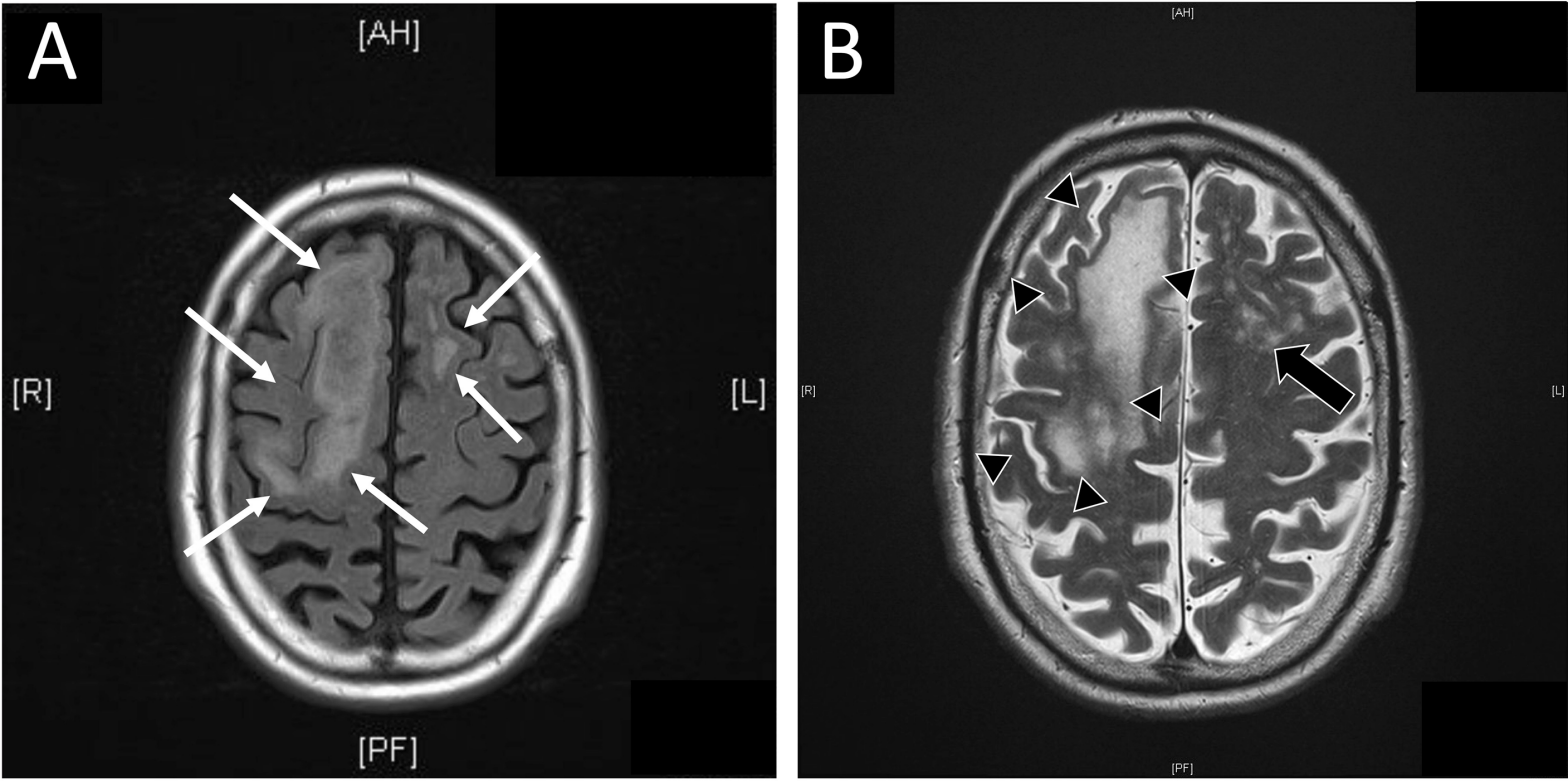
Magnetic resonance (MR) imaging of central nervous system. **(A)** Additional MR imaging was performed as advised after initial cranial computed tomography (cCT). In FLAIR weighted MR diffuse, subcortical located, edematous alterations of cerebral tissue were present (white arrows). **(B)** MR imaging showed subcortical located confluent (black arrow heads) or diffuse (black arrows) signal alterations in T2 weighted image in concordant locations reported in cCT. In opposite to CT, no hint for metastases was reported. Diffuse alterations of subcortical tissue are commonly seen in T2 or FLAIR weighted images in progressive multifocal leukoencephalopathy.

Seven months after initiation of therapy, the patient was admitted to hospital with first onset of latent paresis (left hand, right foot, right facial paresis) and non-fluent aphasia. Apart from these symptoms, no further cognitive impairment- was clinically evident. CT scan of the neurocranium showed small central cerebral lesions accompanied by perifocal edema. Due to the patient’s malignancy, cerebral metastases were (retrospectively wrongly) assumed and anti-edematous treatment with dexamethasone (12 mg/d) was initiated. Additionally, in further work-up performed MR imaging did not show central lesions, but unspecific subcortical alterations were observed in T1- and T2-weighted images (**Figures 1A, B**). Electroencephalography detected right frontal delta-/theta-wave patterns and abnormal alpha-shaped ground rhythm both upon admission and in three-week intervals. There was no hint for tumor recurrence or other pathological findings in the thoracal and abdominal CT scans.

Due to deterioration of symptoms including neglect, apraxia and progredient aphasia, the patient was transferred to the department of neurology for further diagnostic workup. Vascular pathology of the carotids was excluded by ultrasound. Cerebrospinal fluid (CSF) comprised normal cell count (1/nl, ULN 5/nl) with discrete lymphomonocytosis and no oligoclonal banding, but elevated tau protein (647 pg/ml, ULN: 450 pg/ml). Analysis of CSF by polymerase chain reaction finally detected JCV (720 copies/ml) and PML was diagnosed. HIV and other viral or bacterial infections were repeatedly ruled out.

**Figure 2:**
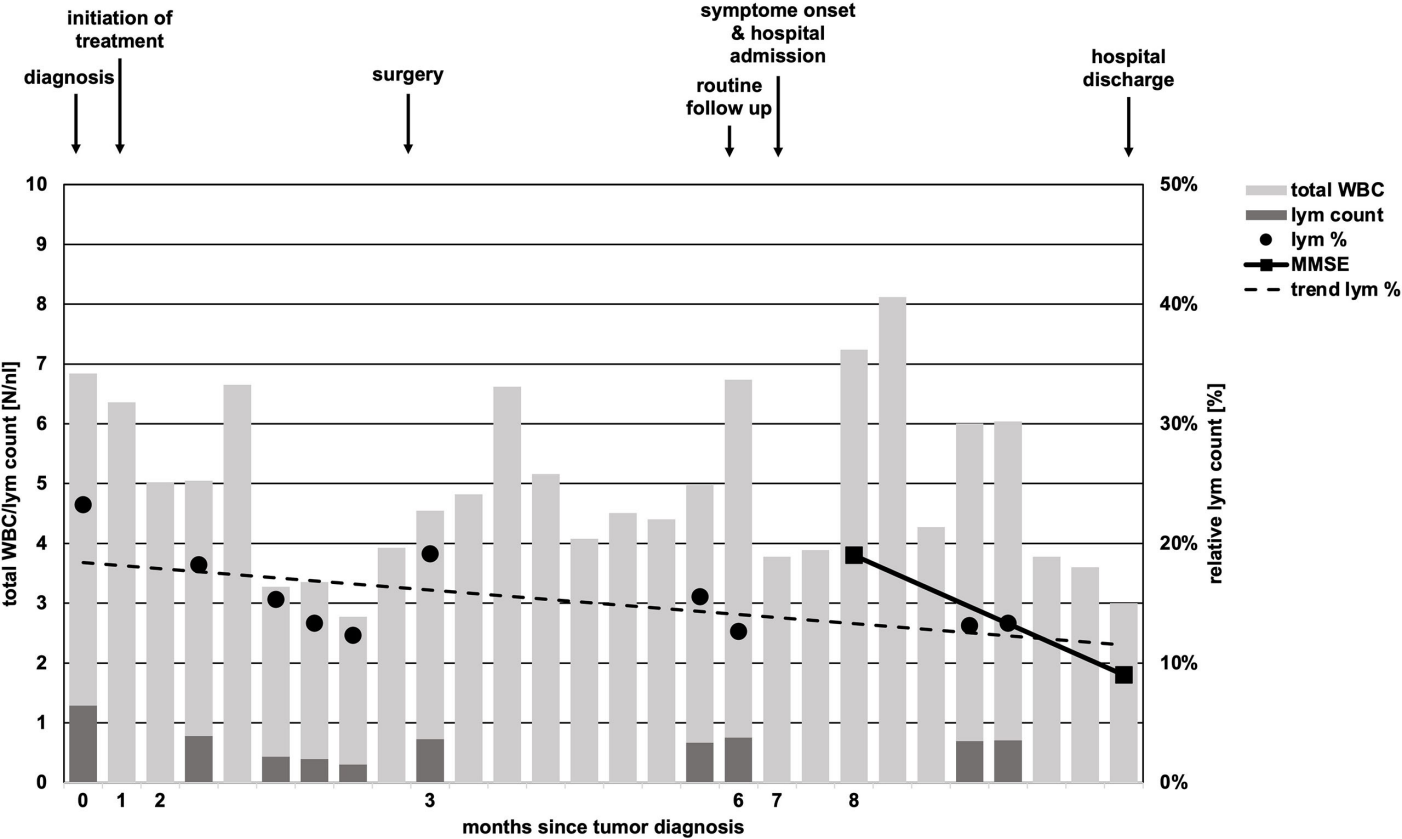
Timeline of total white body cell and lymphocyte count. While total white body cell (WBC) count (light grey bars) undulated in course of disease, absolute lymphocyte (lym) count (dark grey bars) was constantly low and never reached pre-treatment levels in course of time. Relative lymphocyte count (black dots, lym %) showed a trend of decline (scattered black line, trend lym %). Missing black dots/dark gray bars represent missing laboratory parameters regarding lymphocyte counts. Arrows above chart indicate initiation of chemotherapy, time of surgery, follow-up, onset of neurologic symptoms in course of PML and hospital discharge.

Within 6 weeks after initiation of carboplatin and paclitaxel containing chemotherapy, laboratory parameters showed no relevant pathology, besides mildly reduced leukocytes (minimum value of 3.0/nl, normal range 4.0-11.0/nl, **Figure 2**). By differential blood count, persistent lymphopenia of 12.6% (normal range: 20-45%, total lymphocytes: minimum of 300/nl) was observed until death (**Figure 2**). No subdifferentiation of lymphocyte populations was performed.

Upon PML diagnosis, treatment with dexamethasone was immediately discontinued. Therapy targeting PML comprised mirtazapine (30 mg 1x/day), mefloquine (250 mg 3x/day), cidofovir (370 mg/every two weeks), vitamin b12 and folic acid as proposed in the literature (9). However, there was no relevant relief in neurologic symptoms over the following weeks and Mini–Mental State Examination score gradually declined (19 to 9 points). Physiotherapy and speech and language therapy were performed but showed no relevant improvement in symptomatic burden. Despite the short duration of treatment up to this point, the above-mentioned agents were discontinued according to the patient’s will after 2.5 weeks. To limit further neurologic deterioration, a rehabilitation program was arranged. However, the patient lost his mobility and developed further clinical decline within a few weeks and palliative care was finally initiated. The patient died two months after the onset of first symptoms.

## Discussion

Mechanisms of JCV reactivation and consecutive development of PML are just partly understood (10). Impaired viral control due to alterations in lymphocytes – either by reduction of absolute lymphocyte counts or impaired lymphocytic function – are assumed (11, 12). While PML is commonly observed in HIV-positive individuals without antiviral therapy, it is uncommon in patients undergoing sufficient HIV-directed therapy underscoring the relevance of adequate lymphocyte count and function (13, 14). In hematological malignancies, lymphocyte function and/or number are either compromised by the disease itself (e.g., in lymphomas) or because of its treatment (15, 16). Numerous cases and some studies have been published in the context of chronic autoimmune diseases (inflammatory bowel diseases or rheumatologic diseases) requiring immunosuppressive therapy (17). Because of these acquired forms of immunodeficiency, affected individuals are prone to JCV reactivation.

**Figure 3:**
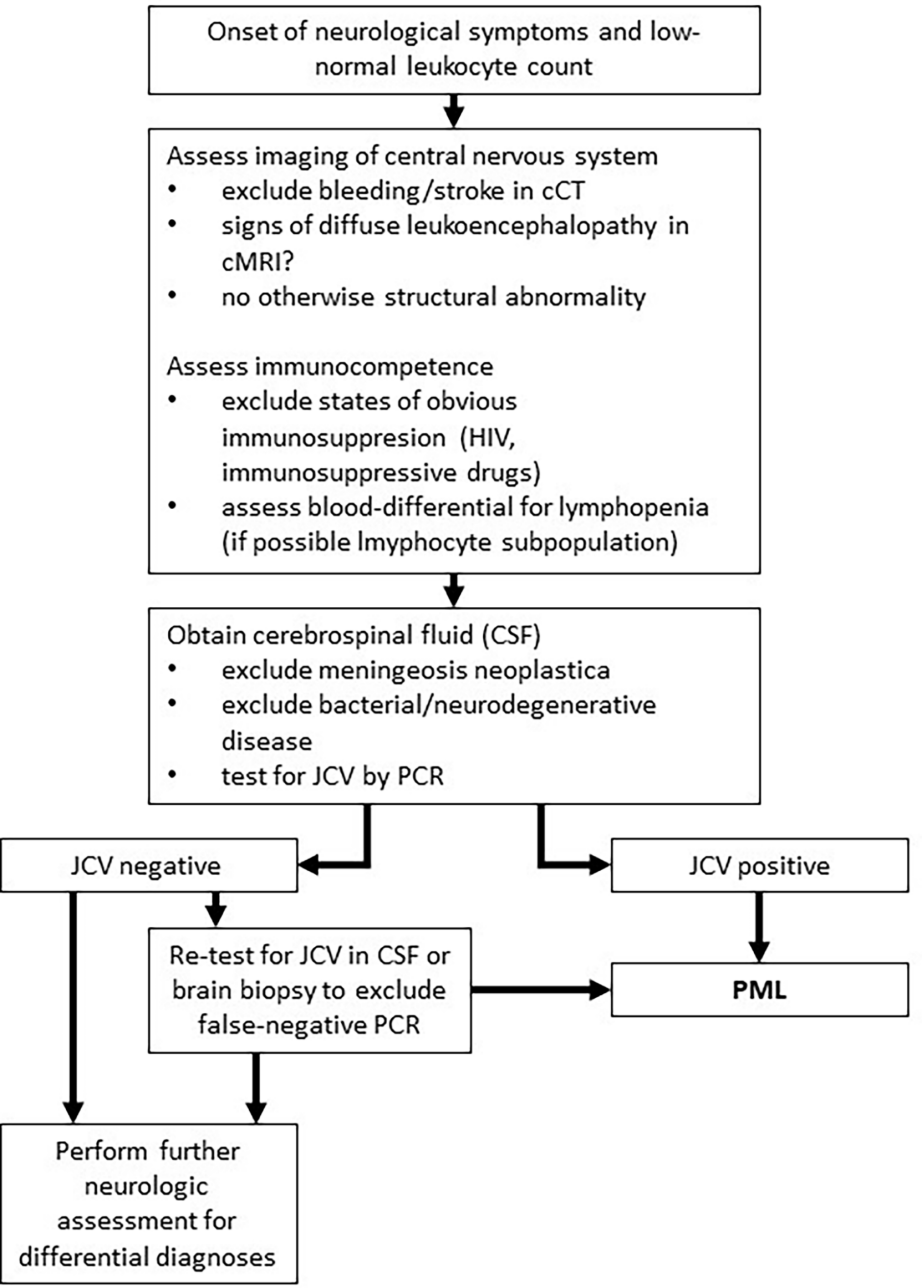
Adopted algorithm for PML diagnosis in solid tumors. This algorithm represents a potential approach to PML diagnosis for patients with solid tumors based on literature research (28).

This case highlights a disease history of PML development in a patient without an obvious prolonged immunosuppressive state. Diabetes mellitus is associated with increased risk of infections, however, up to now just one case of PML in an individual suffering from sole diabetes mellitus without additional risk factors is reported in the literature (18). The subsequent antineoplastic therapy for esophageal cancer was the only obvious immunosuppressive factor in our patient (19). In solid tumors, only scattered case reports of PML in otherwise healthy individuals are published. Lymphopenia was the only tangible risk factor in our case. The persistent lymphopenia was striking (**Figure 2**) and the total lymphocyte count was lowered to AIDS-like levels, suggesting relevant impairment of immunocompetence. Unfortunately, determination of lymphocyte subdifferentiation was not performed in routine clinical practice. Absolute and relative lymphopenia occurred specifically after administration of a taxane- and platin-based chemotherapy. A study performed by Verma *et al.* demonstrated a delayed and (with respect to distribution subsets) altered lymphocytic repopulation in breast cancer patients undergoing taxane- and platin-based systemic chemotherapy (20). Thereby, B cells and CD4^+^ T cells were significantly differentially subdistributed after repopulation, suggesting impairments of immunologic capabilities. In contrast, Waidhauser *et al.* showed that chemotherapy causes alterations in B cells, while T cells were not altered (21). PML was also reported in a case of ovarian cancer after treatment with carboplatin and paclitaxel and in one case of lung cancer after having received carboplatin and gemcitabine (5, 6). In both cases, authors reported transient lymphopenia. Whether PML is at least in part a phenomenon of certain substances of systemic therapy or merely the consequence of lymphopenia caused by chemotherapy remains unclear. Increasing evidence demonstrates that chemotherapies can cause changes of the cellular immune response by different mechanisms (21–23). These “side effects” are used in the context of immuno-chemotherapies apart from cytotoxic effects. Immunotherapies are known to cause some exhaustion and misregulation in the context of tumor therapy (24). This situation, in addition to the undoubtedly clinically leading lymphopenia, might further increase the risk of JCV reactivation. While overstimulated immune response is routinely treated by corticosteroids and neutropenia is well amendable by stimulation with granulocyte colony stimulating factor, there is no established treatment option for lymphopenia or disrupted lymphocyte function, yet. However, some authors report PML remission under checkpoint inhibitor therapy (25, 26). High rates of leukopenia and accompanying lymphopenia in the treatment of solid tumors with conventional systemic agents are routinely observed (27). This causes a state of, usually time-limited, immunosuppression and alterations in immune response, which lacks therapeutic strategies regarding lymphopenia. This transient state of immunosuppression leads to a potentially significant underestimated risk of JCV reactivation in the long-term. Thus, solid tumors undergoing conventional chemotherapy need increased awareness of the rare, but nevertheless serious reactivation of JCV and consecutive fatal PML. Additionally, PML has to be at least considered in neurologic deteriorating patients with either known or assumed/non-excludable states of immunosuppression. **Figure 3** shows an adopted, possible algorithm for PML differential diagnosis in patients suffering from solid tumors. With respect to the rather disappointing therapeutical options (an overview of currently discussed treatment strategies is provided in **Table 1**). Awareness and early detection of PML is crucial to prevent further harm for patients.

**Table 1:** Overview of currently discussed treatment options in PML.

Treatment approach	Mechanism of action	Outcome (Source)
Cytarabin	Nucleotide analogue	No improvement in survival rate compared to no cytarabin (p=0.85) (29)
Cidofovir	Nucleotide analogue	Decrease of survival in cidofovir group (30) No improvement in cidofovir group (HR 0.93, CI 0.66-1.32) (31)
Topotecan	Inhibitor of topoisomerase I	Treatment response in 3 of 12 patients, hematologic side effects (32)
Mirtazapine	Invasion block of JCV into oligodendrocytes by antagonism of 5HT2/5HT3 serotonin receptors	Anecdotal improvements in several cases, mostly in combination with antiviral therapy in HIV-positive individuals (33–38)
Mefloquine	Unknown	*In-vitro* inhibition of JC Virus replication, no benefit in DNA load *in vivo* (39, 40)
Interleukin-7	Immunostimulation to increase lymphocyte count	Increase in lymphocytes and decrease in JCV viral load, but no clear improvement in 1-year-survival rate (54.7%) (41)
Filgrastim	Granulocyte colony stimulation factor	100% survival 2 years after PML onset, retrospective study (42)
Checkpoint inhibitor (CPI)	Upregulation of activity of cytotoxic T cells	Improvement in up to 62.5% of cases, but case of PML onset under CPI therapy (26, 43)
Autologous or allogeneic T cells	HLA-matched transfer of immunocompetent T cells	Survival rates of up to 67% (44, 45)

According to UpToDate (as of June 16th 2022) following treatment approaches are theoretically considered for PML therapy because of hypothetical mechanisms in small numbers of patients, but without clear proof of efficiency, whereof most agents have been studied in HIV positive individuals (46).

The limitations of our case report are the single case character of this report, the partially incomplete laboratory diagnostics due to clinical routine and its retrospective approach.

The strength of our report is the demonstration of a highly relevant and in clinical routine presumably completely underestimated complication - PML - in the context of conventional systemic therapies in solid tumors. To the best of our knowledge, we report the first case of PML in esophageal carcinoma in an immunocompetent individual. Furthermore, regarding the other cases of PML in solid tumors reported so far, a first attempt is made to outline mechanistic correlations and pathophysiological causes.

## Conclusion

In contrast to HIV-positive individuals or patients suffering from hematological malignancies, JCV reactivation and consecutive PML is an extremely rare event in solid tumors. Chemotherapy-induced and prolonged lymphopenia is a relevant risk factor for JCV reactivation. These circumstances carry the risk of potentially overlooked PML-diagnosis - especially in view of a presumably obvious metastasis of the central nervous system. Since therapeutical options addressing lymphopenia are missing, patients need critical evaluation of differential blood counts before therapy onset and thorough monitoring during and between therapies. Additionally, neurologic symptoms in patients suffering from solid tumors undergoing treatment need to be thoroughly evaluated and PML must be considered as a possible complication.

## Data availability statement

The data analyzed in this study is subject to the following licenses/restrictions: The reported case of progressive multifocal leukoencephalopathy occurred as a SUSAR in course of the ESOPEC trial as stated within the manuscript. Requests to access these datasets should be directed to Jens.Hoeppner@uksh.de.

## Ethics statement

The reported case occurred as a SAE in course of the ESOPEC trial. The ESOPEC trial was reviewed and approved by the ethics committee of the Albert- Ludwigs University Freiburg (315/15FF-MC). The patients/participants provided their written informed consent for study participation.

## Author contributions

PM, ML, RC: study design, data acquisition, data interpretation, manuscript drafting; MS, JH: data acquisition, critical review of manuscript; FL-S, JS: diagnostics, manuscript revision; JG: clinical treatment of patient, data interpretation, manuscript drafting. All authors contributed to the article and approved the submitted version.

## Conflict of interest

The authors declare that the research was conducted in the absence of any commercial or financial relationships that could be construed as a potential conflict of interest.

## Publisher’s note

All claims expressed in this article are solely those of the authors and do not necessarily represent those of their affiliated organizations, or those of the publisher, the editors and the reviewers. Any product that may be evaluated in this article, or claim that may be made by its manufacturer, is not guaranteed or endorsed by the publisher.

## Abbreviations

JCV, JC virus; PML, progressive multifocal leukoencephalopathy; HIV, human immunodeficiency virus; (c)CT, cranial computed tomography (of neurocranium); AEG, adenocarcinoma gastro-esophageal junction; NCT, national cancer trial; ESOPEC, name of clinical study in esophageal cancer treatment; FLOT, chemotherapy acronym: fluorouracil, leucovorin, oxaliplatin, taxane; CROSS, name of clinical study in esophageal cancer treatment; Gy, Gray (radiation dosage); HbA1c, hemoglobin A1c; ULN, upper limit of normal; CT, computed tomography; MR, magnetic resonance; CSF, cerebrospinal fluid; AIDS, acquired immunodeficiency syndrome; CD, cluster of differentiation; GCSF, granulocyte colony stimulating factor.
